# Restoring Occlusion in Mutilated Arches

**Published:** 1914-04-15

**Authors:** Henry W. Gillett


### RESTORING OCCLUSION IN MUTILATED ARCHES.

BY HENRY W. GILLETT, D. M. D.

I now desire to take up the class of cases where we are likely to have all the conditions due to early mutilation, plus the loss of so much of the tissue adjacent to the teeth from the ravages of pyorrhea, as to result in areas between the teeth and possibly between the roots of molar teeth, impossible with any feasible and reasonable expenditure of time and effort for the patient to keep in a condition that will not invite the return of active pyorrhea, and that will not be a menace to his welfare on the score of insanitation.

We will assume that the active pyorrhea has been eliminated by curing where feasible, and by removal of all teeth where the lesions had been so extensive as to demand that procedure.

Such being the case, let us spend a moment in summing up the situation and in considering the ends we desire to attain. In our typical extreme case we have a mutilated organ in which the remaining teeth have been weakened as regards attachment to the jaw, by the inroads of disease. Therefore, our *first* aim is to conserve all the remaining strength of each tooth deemed worthy of retention; our *second* is to do this along lines that shall not only permit, but shall promote the maintenance of continuous, healthy conditions by the patient at every point, but particularly at the gum margins; and *third*, we desire to restore an occlusion that will be efficient for the thorough mastication of food.

Under the conditions of our typical case, health of remaining natural tissues and organs and efficiency of the masticating organ as a unit have become the paramount issues—they overshadow all considerations of sentiment, as to the retention of natural organs which shall interfere with their realization.

Just as teeth which have become so diseased as to be a permanent menace to the health of the subject should be removed, so portions of the teeth which gravely menace continuance of the restored local health or which prevent realization of efficiency of function in the organ of which they are parts, must be treated in the same way.

Let us consider a case where clearing away of the hopelessly diseased parts has left but a few teeth in either or each jaw, either scattered about or the six front teeth standing together, where there is a pronounced tendency to the collection of sordes, and where the absence of the septa and the relation of the teeth prevent efficient cleaning by the patient. In such a case is there any hope that the three objects enumerated above; conservation of all remaining strength maintenance of continuous health of the soft tissues, and restoration of occlusal efficiency, can be attained and maintained so long as the crowns of the teeth in such a jaw remain in situ to interfere with the patient's cleaning efforts, and by the malrelations certain to exist, prevent the fruition of the prosthodontists efforts?

My own answer is that there is no such hope; and with the courage of my convictions I have proceeded to cut to the gum level all the teeth in jaws in such a state as I have described. After this has been done, the neatest possible fitting copes are prepared for each root end. These copes are united either in sets of two or more (often all the copes in one jaw are united together) and the selected measures for retention of the removable bridge pieces are mounted on the resultant appliance.

By this procedure I have succeeded in several cases in attaining all the three desired objects above enumerated.

The strength of each individual root and of all the roots collectively has been conserved by uniting them so they get mutual support, and by using a removable appliance the leverage stress upon them has been materially reduced.

I desire to direct your attention more particularly to both the points mentioned in the last sentence. You will note that by the procedure indicated, all the advantages claimed for fixed bridge work as regards mutual support and fixation of individual roots have been realized. I am not sure whether this difference in leverage stress has received attention in our literature, but it is my contention that aside from the greater latitude frequently given the operator in the arrangement of the occlusal planes in a removable piece, there is a further advantage in the shortening of the lever through which the stress is directly applied to the root attachments, and from the fact that the joint between the fixed and removable parts of the bridge serves to ease the lateral trust upon the roots.

A condition has thus been established leaving no excuse for inefficiency in the cleaning operation of the patient because the brush and other cleaning devices can be applied directly to the weak point in all incipient pyorrhea, the gum margin where the lesions begin. Furthermore, all the gingivae can receive the vigorous friction so valuable in stimulating a healthy blood supply. Some qualification is perhaps desirable here to allow for the fact that the bars uniting the copes cover a limited area of gum so as to render it less accessible to the brush. In practice I do not find this a disturbing factor.

By the described procedure, there has been eliminated from the jaw under consideration every impediment hindering the prosthodontist in the establishment of normal occlusal lines in that jaw.

I am aware that this suggestion of cutting possibly sound teeth to the gum level will seem radical to many of you, and I anticipate that it may stir some of you to drastic criticism; but my experience with them has been so satisfactory that my faith in the value of the principles under-lying the procedures described has grown stronger with each successive case.

After two years of practical service, I am able to say that nothing in my professional career has given me greater satisfaction than its entire success in realizing all the three ends I have emphasized.—*January Dental Summary.*

